# Human Visceral Leishmaniasis in Brazil in the Past 20 Years: An Epidemiologic Update

**DOI:** 10.1590/0037-8682-0019-2025

**Published:** 2025-10-17

**Authors:** Fernanda Moreau de Almeida Soares, Thiago Soares Rocha, Everton Rusciolelli Nascimento, Gisele Lopes de Oliveira, Marcio Bezerra Santos, Sebastião Rodrigo Ferreira

**Affiliations:** 1 Universidade Federal do Sul da Bahia, Centro de Formação em Ciências da Saúde, Teixeira de Freitas, BA, Brasil.; 2 Universidade Federal de Alagoas, Centro de Ciências Médicas e Enfermagem, Arapiraca, AL, Brasil.

**Keywords:** Visceral Leishmaniasis, Neglected disease, Zoonoses, Socio-environmental determinants

## Abstract

**Background:**

Human visceral leishmaniasis (HVL) is a neglected tropical disease that remains highly lethal if left untreated and continues to affect public health*.* In Brazil, *Leishmania infantum* is the main etiological agent, and domestic dogs are considered the primary reservoir. Considering the historic importance of leishmaniasis in Brazil, we aimed to gather information on the epidemiology of HVL in Brazil over 20 years.

**Methods:**

We conducted an integrative review through a bibliographic survey of articles published between 2002 and 2022.

**Results:**

The process resulted in the inclusion of 75 studies. Most of these studies reported data from the northeastern region, which continues to present the highest incidence rates nationwide. Most cases were concentrated in men and children under 10 years of age, particularly in urban and peri-urban areas. Although national case numbers have recently declined in Brazil, the persistence of endemic areas, driven by poverty, inadequate sanitation, deforestation, and unplanned urban growth, underscores the ongoing public health relevance of HVL.

**Conclusions:**

The ongoing spread of HVL and variation in its occurrence across regions underscore the unequal impact of the disease. HVL is not only a parasitic infection but also a manifestation of broader structural inequalities.

## INTRODUCTION

Human visceral leishmaniasis (HVL) is a neglected tropical disease with worldwide distribution. It is zoonotic in nature and more prevalent in populations with greater socioeconomic vulnerability. The disease is present in more than 70 countries, and although approximately 50,000 to 90,000 new annual cases of HVL occur globally, only 25%-45% are properly reported to the World Health Organization. Brazil, India, and East Africa account for 90% of all the HVL cases worldwide^1^ ^-^ ^3^ .

HVL is caused by intracellular protozoans of the *Leishmania* complex*.* In Brazil, the etiological agent is *Leishmania infantum*, and the primary vector in urban areas is *Lutzomyia longipalpis*, also known as “sandfly.” Importantly, in urban environments, the main reservoirs of *L. infantum* are dogs (*Canis familiaris*), which have zoonotic characteristics^4^.

In humans, the infection has an incubation period between 10 days and 24 months, with an average of 2-6 months. The main clinical signs and symptoms of HVL include persistent fever, weight loss, hepatosplenomegaly, jaundice, hemorrhage, dyspnea, diarrhea, bacterial infections, and hematological impairments, such as thrombocytopenia and severe neutropenia^5^. If left untreated, it can lead to death from the disease itself or to hemorrhagic complications in approximately 90% of cases^6^ ^,^ ^7^.

In the 1990s, approximately 90% of the reported cases of HVL in Brazil were recorded in the northeastern region, typically in rural areas. As the disease spread to other regions, the situation changed, and in 2012, the northeast region accounted for 43.1% of the country's cases. Meanwhile, the number of cases increased in the southeast, and the disease became predominantly urban^7^. 

The urbanization of HVL remains unclear. However, adaptations of *Lu. longipalpis* to temperature variations, peridomicile migratory movements, domestication, proximity to animals, environmental changes caused by human activities, unplanned urban occupation, and exploitation of the wild environment have contributed to disease urbanization^6^ ^,^ ^7^.

HVL is a compulsory notifiable disease in Brazil according to the Notifiable Diseases Information System (*Sistema de Informação de Agravos de Notificação* [SINAN]), and every suspected case must be notified and investigated. The incompleteness of the epidemiological data on HVL indicates that official records underestimate the actual prevalence of the disease. The absence of integration between case and environmental surveillance services (relating to the vector, reservoirs, and transmission areas) has resulted in a fragmented database of HVL across territorial units at different political-administrative levels^8^ ^,^ ^9^, making it difficult to build policies and control the disease. Review studies are essential for understanding the spatial distribution and progression of HVL as well as the socioenvironmental factors involved in its transmission and persistence.

This study aimed to conduct an integrative review of the scientific literature and characterize the epidemiological profile of HVL in Brazil from 2002 to 2022. By analyzing epidemiological surveys and studies based on secondary data, this review examined the geographic distribution of HVL, identified the most affected population groups, assessed temporal trends, and explored the key factors contributing to the persistence of the disease in different regions of the country.

## METHODS

### Study design

This study presents an integrative review encompassing epidemiological surveys and investigations based on secondary data from 2002 to 2022 to elucidate the epidemiological landscape of HVL in Brazil. Most of the analyzed data were derived from the Brazilian Health SINAN, which systematically registers confirmed cases using standardized immunological or parasitological diagnostic methods. 

Immunological diagnosis consists of the detection of anti-*Leishmania* antibodies, which is conducted by Brazil’s *Sistema Único de Saúde* using two techniques: the indirect immunofluorescence reaction test and immunochromatographic rapid test. For parasitological diagnosis, the amastigote forms of the protozoan were obtained from biological samples.

### Data sources and eligibility criteria

The PubMed, Scientific Electronic Library Online (SciELO), and Virtual Health Library (VHL) databases including Medline and LILACS were searched. Controlled descriptors were used according to the Descriptors in Health Sciences and PubMed Medical Subject Headings. The descriptors used were “leishmaniose visceral humana,” “calazar,” “epidemiologia,” and “Brasil” in Portuguese and “human visceral leishmaniasis,” “kala-azar leishmaniasis,” “epidemiology,” and “Brazil” in English. The boolean operators “AND,” “OR,” and “NOT” were applied, the latter particularly for the descriptor “review” in the PubMed database. The search strategy is shown in Supplementary File 1.

The inclusion criteria were as follows: (1) written in Portuguese or English; (2) free full access or available in Periódicos CAPES (a Brazilian database that provides open access to articles for research institutions); (3) no restrictions regarding patients’ age, race, and sex; (4) the keywords appeared in the title or abstract; (5) no direct mention of Brazil in the title or abstract but a mention of a Brazilian city or state; (6) publication and data collection were from 2002 to 2022); and (7) full text was available. 

The exclusion criteria were as follows: (1) reviews, theses, dissertations, monographs, editorials, letters to the editor, forums, comments, author correction, case studies, and books; (2) studies with overlapping or duplicate data; (3) abstract-only papers; (4) studies with animals; (5) entomological and protozoological studies; (6) studies with a restricted population (focused only on children, older people, indigenous people, hospitalized people, people living with HIV, or any other specific group of people or association with specific health conditions); (7) studies on diagnosis, control, and/or treatment methods; (8) risk analysis studies and studies on other diseases; (9) international studies; (10) studies on cost and education; (11) immunology and/or genetic studies; (12) study on mathematical models for disease spread; (13) studies on mortality and lethality; (14) comparison between disease reporting systems; and (15) studies that do not cover the entire municipality.

The retrieved articles were reviewed for compliance with the eligibility criteria by three reviewers. Data from the included articles were extracted by two authors. The extracted data included the first author’s name, year of publication, state, number of HVL cases, period evaluated in the survey, and the most affected sex and age. The articles were arranged in a table, separated according to the Brazilian regions and states in which the research was conducted. Thus, the articles were fully evaluated to describe the epidemiological situation in each state and Brazil as a whole.

## RESULTS AND DISCUSSION

**FIGURE 1:**
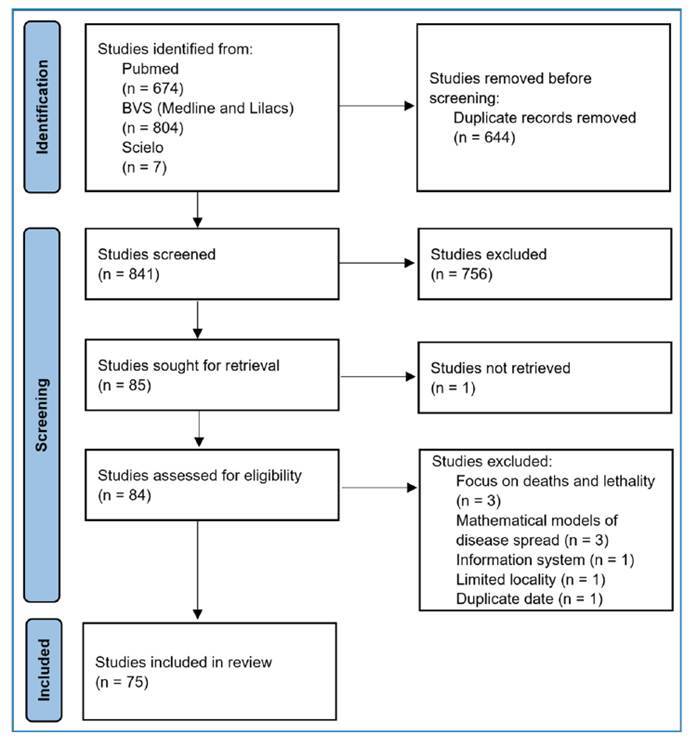
Study selection flowchart.

A total of 76 articles were included **(** Figure 1 **)**. Human visceral leishmaniasis has been reported in 16 Brazilian states (Table 1). Four studies provided nationwide data. In the study by Lopes et al. (2023)^10^, the data specifically referred to Brazil’s border regions. Although the data could not be aggregated owing to overlapping study periods, the study with the longest timeframe, conducted by Azevedo et al. (2019)^11^, reported approximately 53,715 cases over a 16-year period.

**TABLE 1:** Epidemiological studies of human visceral leishmaniasis (HVL) in Brazil, 2002-2022.

Locality	Year	Study Design	Cases	Most Affected Sex and Age	Reference
Brazil	2001-2014	Descriptive study	47,859 notified	Male, <4 years	Reis et al. (2017ª)
	2001-2017	Ecological study	53,715 notified	Not provided	Azevedo et al. (2019)
	2006-2015	Ecological study	37,411 notified,	Male, <1 year	Graepp-Fontoura et al. (2020)
			Ecological study		
	2009-2017	Ecological study	628 notified	Male, 1-4 years	Lopes et al. (2023)
NORTHEAST					
Northeast	2007-2017	Cross-sectional study and spatial analysis	21,703 notified	Male, <10 years	Machado et al. (2020)
São Luís, Maranhão	2002-2010	Cross-sectional study	834 notified	Not provided	Viana et al. (2011)
	2004-2006	Descriptive study	428 notified	Male, <10 years	Silva et al. (2008)
	2007-2016	Mixed ecological and cross-sectional study	415 notified	Male, <10 years	Nogueira et al. (2021)
Maranhão	2000-2009	Ecological study	5,389 notified	Not provided	Furtado et al. (2015)
Fortaleza, Ceará	2001-2006	Cross-sectional study	1,379 notified,	Male, 1-4 years	Albuquerque et al. (2009)
			990 confirmed		
	2007-2017	Ecological study	1,660 confirmed	Male, 20-49 years	Almeida et al. (2020)
Sobral, Ceará	2007-2019	Ecological study	316 confirmed	Male, 1-4 years	Cavalcante F et al. (2022)
Ceará	2003-2017	Ecological study	6,181 confirmed	Male, >5 years	Cavalcante F et al. (2020)
	2007-2011	Descriptive study	annual average 596.8 ± 29.6 cases	Male, 1-4 years	Cavalcante and Vale (2014)
	2007-2018	Cross-sectional study	6,066 confirmed	Male, 20-50 years	Cavalcante K et al. (2022^a^)
	2007-2021	Ecological study	6,775 confirmed	Male, 20-49 years	Cavalcante K et al. (2022^b^)
Teresina, Piuaí	2001-2006	Ecological study	756 notified	<5 years	Almeida et al. (2011)
Piuaí	2007-2019	Ecological study	2,521 confirmed	Male, <10 years	Chaves et al. (2022)
	2007-2017	Ecological study	2,447 notified,	Male, <10 years	Ferreira et al. (2021)
			2,119 confirmed		
	2008-2018	Ecological study	2,492 notified	Male, <10 years	Batista et al. (2021)
Paraíba	2012-2017	Ecological study	1,524 notified,	Not provided	Silva AB et al. (2021)
			327 confirmed		
Alagoas	2007-2013	Ecological study	231 confimed	Male, 0-11 years	Rocha et al. (2018)
	2002	Ecological study	200 notified,	Male, 1-4 years	Oliveira and Montoni (2003)
			134 confirmed		
	2008-2017	Cross-sectional study	352 notified	Male, 1-4 years	Ferreira et al. (2022)
	2007-2018	Ecological study	489 notified	Male, 1-4 years	Braz et al. (2021)
Aracajú, Sergipe	1999-2008	Ecological study	192 notified	Male, 1-4 years	Góes et al. (2012)
	2008-2014	Ecological study	159 notified	Male, 20-49 years	Campos et al. (2017)
Sergipe	2009-2017	Ecological study	556 confirmed	Male, 20-39 years	Almeida et al. (2021)
Caruaru, Pernambuco	2005-2010	Ecological study	29 notified	Not provided	Souza et al. (2014)
Petrolina, Pernambuco	2007-2013	Ecological study	69 notified	Male, 1-4 years	Araujo et al. (2016)
Pernambuco	2002	Cross-sectional study	104 confirmed	Not provided	Aguiar et al. (2003)
	2006-2015	Cross-sectional study	907 confirmed	Male, 1-4 years	Sousa et al. (2018)
	2007-2017	Ecological study	1,186 notified	Male, ≤15	Andrade et al. (2020)
	2014-2018	Ecological study	858 notified	Male, <10 years	Machado et al. (2021)
Petrolina, Pernambuco	2010-2016	Ecological study	107 confirmed	Male, <15 years	Diniz et al. (2018)
			(Petrolina - PE)		
Juazeiro, Bahia			74 confirmed		
			(Juazeiro - BA)		
Barreiras, Bahia	2007-2017	Cohort study	92 notified	Male, 1-4 years	Lacerda et al. (2021)
Natal, Rio Grande do Norte	1990-2014	Ecological study	718 notified	Male, <5 years	Lima I et al. (2018)
Rio Grande do Norte	1990-2014	Ecological study	3,252 notified	Male, 0-4 years	Lima A et al. (2017)
MIDWEST					
Varzea Grande, Mato Grosso	1998-2007	Cohort study	48 notified	Male, <1 year	Missawa and Borba (2009)
Pantanal, Mato Grosso	2007-2016	Ecological study	10 notified	Male, 0-10 years	Brito et al. (2019)
Rondonópolis, Mato Grosso	2011-2016	Cross-sectional study	117 notified	Male, 0-4 years	Luz et al. (2018)
Jaciara, Mato Grosso	2003-2012	Ecological study	19 confirmed	Male, <7 years	Brito et al. (2014)
Mato Grosso	1998-2005	Ecological study	138 notified	Male, <10 years	Mestre and Fontes (2007)
	2002	Ecological study	8 confirmed	Male, 5-9 years and >20 years	Duarte (2003)
Campo Grande, Mato Grosso do Sul	2001-2006	Ecological study	577 notified	Male, <10 years	Botelho and Natal (2009)
	2002-2006	Ecological study	568 confirmed	Male, 0-4 years	Furlan (2010)
	2002-2009	Ecological study	951 notified	Male, <10 years	Brazuna et al. (2012)
	2001-2018	Ecological study	1,855 notified	Male, <5 years	Oliveira et al. (2020)
Três Lagoas, Mato Grosso do Sul	2000-2003	Ecological study	149 confirmed	Male, ≤4 years	Oliveira et al. (2006)
Mato Grosso do Sul	2002-2015	Ecological study	3,137 notified	Not provided	Silva Neto et al. (2020)
	2001-2018	Ecological study	3,566 notified	Not provided	Neitzke-Abreu et al. (2022)
Federal District	2004-2015	Ecological study	321 confirmed	Not provided	Silva et al. (2018)
NORTH					
Cametá, Pará	2007-2016	Ecological study	210 confirmed	Male, <12 years	Miranda C et al. (2021)
Carajás Integration Region	2011-2020	Mixed ecological and cross-sectional study	685 confirmed	Male, 0-5 years	Miranda C et al. (2022)
Araguaína, Tocantins	2007-2012	Ecological study	1,096 confirmed	Not provided	Toledo et al. (2017)
Tocantins	2007-2014	Ecological study	2,885 notified	Not provided	Reis et al. (2019)
	2007-2010	Ecological study	1,779 notified	Male, <10 years	Albuquerque et al. (2014)
SOUTHEAST					
Belo Horizonte, Minas Gerais	2003-2013	Ecological study	866 confirmed	Male, 0-5 years	Bruhn et al. (2018)
Diamantina, Minas Gerais	2016-2018	Cross-sectional study	8 notified	Male, 0-9 years	Batista-Santos et al. (2021)
Paracatu, Minas Gerais	2007-2010	Ecological study	128 notified	Male, 0-14 years	Oliveira and Pimenta (2014)
Montes Claros, Minas Gerais	2007-2009	Mixed ecological and cross-sectional study	95 notified	Male, 0-9 years	Prado et al. (2011)
	2010-2020	Cross-sectional study	413 notified	Male, <19 years	Silva F et al. (2021)
Governador Valadares, Minas Gerais	2008-2015	Cross-sectional study	844 notified	Male, <5 years	Alves and Fonseca (2018)
Araçuaí, Minas Gerais	2012-2017	Ecological study	68 confirmed	Not provided	Cruz et al. (2022)
	2007-2013	Ecological study	41 notified	Male, <4 years	Ursine et al. (2016)
North of Minas Gerais	2007-2011	Ecological study	525 notified	Male, ≤10 years	Gusmão et al. (2015)
Minas Gerais	2002-2013	Ecological study	5,778 notified	Not provided	Silva et al. (2017)
Araçatuba, São Paulo	1999-2015	Ecological study	315 confirmed	Male, 0-4 years	Bermudi et al. (2018)
Adamantina, São Paulo	2004-2011	Ecological study	83 confirmed	Both, 0-4 years	Cardim M et al. (2015)
	2004-2018	Ecological study	352 confirmed	Both, 0-9 years	Rancan et al. (2020)
Bauru, São Paulo	2004-2012	Case series	381 notified	Male, ≤10 years	Ortiz et al. (2015)
	2003-2008	Ecological study	239 confirmed	Male, 0-4 years	Souza et al. (2012)
Birigui, São Paulo	1999-2012	Ecological study	156 confirmed	Both, 0-4 years	Vieira et al. (2014)
Western region, São Paulo	2000-2018	Ecological study	537 notified	Not provided	Santana et al. (2021)
São Paulo	1999-2013	Ecological study	2,324 notified	Male, 0-4 years	Cardim M et al. (2016)

Considering the body of literature (Table 1), there is an observable trend of decreasing the total case numbers (59%); however, the disease has spread to new regions. According to Maia-Elkhoury et al. (2008)^12^ and the Pan American Health Organization^13^, Brazil remains responsible for around 90% of the HVL cases in the American continent.

As shown in Table 1, studies indicated a higher prevalence of cases among men and children under 10 years of age, although HVL can affect individuals of all age groups and both sexes. This pattern may be associated with socioeconomic, spatial, occupational, and behavioral factors, such as the greater presence of men in areas with a higher likelihood of contact with the vector^14^ ^,^ ^15^. In the case of children, vulnerability to infection is attributed to the incomplete development of the immune system, often exacerbated by malnutrition, as well as increased contact with animals, making this group more susceptible to the disease^14^ ^,^ ^16^.

A study conducted by Machado et al. (2020)^17^ in northeastern Brazil identified an average annual incidence rate of 3.6 cases of HVL per 100,000 inhabitants, with most cases occurring in urban areas and predominantly affecting children aged <10 years and men. The highest risk of occurrence was observed in municipalities located within the Cerrado and Amazônia biomes and to a lesser extent, in Caatinga. Despite social advancements in the region during the study period, the epidemiological profile of the disease remained unchanged, suggesting that these improvements were insufficient to reduce risk among the most vulnerable populations.

In the state of Maranhão, four articles were selected, with one study focusing on the whole state^18^ and the others on the state’s capital, São Luís^19^ ^-^ ^21^. This state is among the five states with the highest HVL incidence rate in Brazil at 3.32 cases per 100,000 inhabitants by 2022^13^ ^,^ ^22^. HVL was established as an epidemic in São Luís in 1982 and is now classified as an urban endemic focus^20^. Viana et al. (2011)^19^ associated higher HVL notification rates with periods of increased rainfall. However, the authors did not account for the time lag between infection and onset of clinical manifestations of the disease.

The state of Ceará had seven selected articles that covered not only municipal research^23^ ^-^ ^25^ but also statewide research^26^ ^-^ ^29^. Ceará is also among the states with a high incidence rate of 1.8 cases per 100,000 inhabitants in 2022^30^. HVL has been endemic in Ceará since the 1930s, and studies continue to report epidemiological indicators and a high risk of transmission in the state. Migratory flow in the region, combined with socio-environmental factors, contributes to the ongoing spread of the disease^28^. According to Cavalcante and Vale (2014)^25^, although the highest notification rates occur in municipalities with higher human development index (HDI) scores, these cases are likely to originate from poorer areas within these more developed municipalities. 

The state of Piauí had four articles regarding HVL^31^ ^-^ ^34^. The paper published in 2022^31^ stands out because of its more recent data and longer period of analysis (2007-2019). A strong tendency toward endemicity of HVL was observed in Piauí. In 2007, the state accounted for 10.1% of HVL notifications in Brazil, whereas in 2019, this percentage decreased to 9.6%. This small decrease highlights that there has not been significant progress in disease control in the state.

Only one article on Paraíba was included in the review. The study conducted by Silva et al. (2021)^35^ explored the relationship between HVL incidence and socioeconomic factors. A 0.1-point increase in HDI could lead to a 2.6-fold increase in HVL cases, whereas a 1% increase in the proportion of the population living in poverty would result in a 5% increase in the probability of disease occurrence. Paraíba ranks seventh among the ten Brazilian states with the highest proportion of poor residents^36^. These findings underscore the vulnerability of the state and highlight the urgent need for further research on HVL in this region.

Four manuscripts dealt with the state of Alagoas^37^ ^-^ ^40^. A more recent study^39^ showed that 64.7% of the municipalities in Alagoas reported cases of HVL, representing a 10.8% increase in the number of municipalities affected by HVL compared with the study by Rocha et al. (2018)^38^. Similar to Paraíba, Alagoas appears among the 10 poorest states in Brazil, ranking third^36^.

Three articles were selected for Sergipe, two of which focused specifically on the capital, Aracaju^41^ ^,^ ^42^. These studies highlight the endemic nature of HVL in these municipalities. Between 2008 and 2014, Aracaju consistently reported a higher incidence per 10,000 inhabitants than the state average. In 2008, the incidence coefficient in Sergipe was 0.22, compared to 0.34 in Aracaju. The rates were 0.40 for Sergipe and 0.68 for Aracaju in 2010 and 0.30 for Sergipe and 0.50 for Aracaju in 2014^42^. Between 2009 and 2017, 556 HVL cases were confirmed, with an annual average of 62 cases^43^. A predominance was observed among men aged 20-39 years, which differs from the pattern reported in most studies included in this review.

Pernambuco had the highest number of selected articles (seven) in the northeast region, four of which had statewide coverage^44^ ^-^ ^47^. Other studies have focused on isolated municipalities^48^ ^-^ ^50^. HVL is a problem in the state, which is among the 10 states with the highest number of cases, with 1.82 an incidence rate of cases per 100,000 inhabitants^51^. From 2006-2015, the percentage of municipalities with reported cases increased from 21.1% to 43.8%^44^. In 2014, Petrolina had an incidence rate of 6.1 cases per 100,000 inhabitants, which was higher than the national average (1.7 cases per 100,000 inhabitants)^49^. 

Two studies were conducted in Bahia, focusing on the municipalities of Juazeiro and Barreiras. The first was the same study that also analyzed the municipality of Petrolina in the state of Pernambuco^49^. Similar to Petrolina, Juazeiro exhibited a high HVL incidence of 8.6 cases per 100,000 inhabitants, and the sociodemographic characteristics of the affected populations in both municipalities were similar. The comparable incidence rates in these two municipalities may be attributable to their close geographic proximity, being separated only by an approximately 800-meter bridge over the São Francisco River. HVL in Barreiras predominantly affects male children, with a mean age of 4 years. Most cases occurred in urban areas, indicating disease urbanization. This pattern is associated with rapid urban expansion into previously rural zones^52^.

Two studies addressed the occurrence of HVL during the study period in the state of Rio Grande do Norte. Disease distribution is associated with unplanned urbanization and low socioeconomic status. Additionally, a shift in the epidemiological profile was observed, with a reduction in the number of cases among children, which may be related to improved nutritional conditions^53^ ^,^ ^54^.

In the Midwest region, six studies conducted in the state of Mato Grosso^55^ ^-^ ^60^ indicated that HVL has spread across the state. This expansion has been linked to unplanned urban development and migration, resulting in distinct patterns of disease distribution. The occurrence of HVL is sporadic in the Pantanal area, with only 10 cases reported in children. By contrast, Rondonópolis reported 117 cases, underscoring the vulnerability of young children and individuals coinfected with HIV.

Between 2001 and 2018, the state of Mato Grosso do Sul reported 3566 cases of HVL^61^. Consistent with Silva Neto et al. (2020)^62^, HVL incidence increased during La Niña phases and decreased during El Niño periods, reflecting the influence of climatic factors on vector biology. In Campo Grande, the municipality with the highest number of studies, HVL has become endemic^63^ ^-^ ^66^. According to Oliveira et al. (2020)^66^, 1,855 cases were reported in the city over a 17-year period. The disease primarily affects children aged <5 years and men, a pattern that was also observed in the municipality of Três Lagoas^67^. The disease is concentrated in vulnerable urban areas, and a higher risk of occurrence is associated with low socioeconomic and infrastructure indicators^64^ ^-^ ^66^.

Completing the midwest region, one article^68^ addressed the issue of HVL in the Distrito Federal. This work emphasizes that for epidemiological surveillance to function properly, the system requires continuous attention in terms of structure and response capacity, corroborating the discussion in articles on other states in the region ^68^.

In the north, only two states were included: Pará and Tocantins. The states in the north that did not have any article coverage were Rondônia, Acre, Amazonas, and Amapá. This lack of research might be related to the low number of confirmed cases in these federal units, which, according to SINAN data from 2010 to 2021, added to a total of 20 cases^69^. The low population density in the region may be a reason for the low incidence, which is mainly explained by the extent and difficulty of locomotion in the Amazon rainforest and the lack of infrastructure.

Two studies on HVL in Pará^70^ ^,^ ^71^ highlighted deforestation and unsustainable land use in the Amazon as key factors driving the spread of infectious diseases. In the Carajás Integration Region, 685 cases have been reported, mostly among boys aged 0-4 years. HVL incidence significantly increases with the formation of an epidemiological corridor along highways and rivers. Case distribution is linked to human activities, such as urbanization, livestock farming, and mining, as well as deforestation, forest fragmentation, and pasture development. Municipalities with a higher gross domestic product and varying HDI levels also have a high HVL prevalence, indicating that the risk is not limited to the poorest areas^70^.

Among the articles that dealt with Tocantins^72^ ^-^ ^74^, one^73^ showed that the state was responsible for a high number of cases from 2007 to 2014 (2,885 cases), resulting in the northern region overtaking the northeastern region in terms of HVL incidence during this period. The authors associated this increase with the climatic conditions of the state, which is located between the Amazon and the Cerrado, acquiring the characteristics of both biomes. 

Although the environmental conditions in the northern region are favorable to the vector and the socioeconomic characteristics resemble those of the northeast, the population density in the north is significantly lower than that in other regions (4.7 inhabitants per square kilometer).

In the southeast region and across the country, Minas Gerais was the state with the highest number of selected studies, totaling ten, including one addressing the state's overall epidemiological situation, another focusing on the northern region of Minas Gerais,^16^ ^,^ ^75^ and eight focusing on specific municipalities^76^ ^-^ ^83^. In Montes Claros, the most affected municipality, HVL has become an established urban endemic, with 508 cases reported between 2007 and 2020, primarily in male children and adolescents^79^ ^,^ ^83^. Favorable environmental conditions, socioeconomic vulnerability, and migratory flow since the 1970s have contributed to its persistence. In Diamantina (2016-2018), HVL exhibited a rural-to-urban transition profile and high lethality^77^. According to Bruhn et al. (2018),^76^ in Belo Horizonte, social determinants, such as low educational level, older age, black or brown skin color, and limited access to healthcare, were the primary factors associated with elevated mortality from HVL between 2006 and 2013, independent of clinical conditions such as HIV. The case fatality rate remained high at approximately 11%, with an upward trend during the study period^76^. According to the aforementioned studies, the disease is prevalent mostly in urban areas, reflecting the ongoing urbanization of HVL. The disease has spread unevenly throughout the state, predominantly in Central Mineira, Jequitinhonha, the Metropolitan areas of Belo Horizonte, Northwest Minas, North Minas, and Vale do Rio Doce. Most cases occur in boys children aged <10 years.

Eight studies were conducted in São Paulo, and one covered the epidemiological profile of the entire state^14^. In São Paulo, HVL expansion is strongly associated with transportation infrastructure, particularly the Marechal Rondon Highway, which acts as a corridor for vector and disease dispersion^14^. In Adamantina and its micro-region (325 cases, 2004-2018), HVL became endemic, spread along major roads, and affected both central and peripheral urban areas^84^ ^,^ ^85^. In Birigui (156 cases, 1999-2012), cases were initially concentrated in the central zones, but gradually expanded, with girls being more affected among children aged 0-4 years^86^. Between 1999 and 2015, the highest HVL incidence rate in Araçatuba occurred in children aged 0-4 years old. The initial period showed a cyclical and spatially dependent pattern, whereas the later period showed low incidence and random distribution. This shift may have been related to the implementation of control measures^87^. Spatial clusters were associated with close human-animal interactions, organic matter accumulation, and poor socioeconomic conditions. In Bauru and 45 municipalities in western São Paulo (537 cases, 2000-2018), the disease followed mobility routes and was linked to socioeconomic vulnerability and environmental factors^88^ ^-^ ^90^.

As mentioned previously, HVL is a notifiable disease; therefore, every suspected case must be notified before it is confirmed. Confirmed cases were those that met any of the confirmation criteria, which could be clinical laboratory or clinical epidemiological. The clinical laboratory criteria were based on finding the parasite in direct parasitological tests and/or culture, as well as immunofluorescence reactivity with a serological titer of 1:80 or more, excluding differential diagnoses. Compliance requires identification of at least one of these factors. However, the clinical-epidemiological criterion requires the patient to come from an area with HVL transmission without laboratory confirmation, but with a favorable response to the therapeutic test^91^.

As described in Table 1, 56.57% of the articles surveyed considered all notifications in the database without prioritizing case confirmation, 35.52% focused only on confirmed cases, 6.57% displayed the quantities of both notified and confirmed cases, and 1.31% did not specify whether the cases were notified or confirmed. This lack of uniformity limits the accurate determination of the true number of confirmed HVL cases in Brazil. According to the Brazilian Ministry of Health (*Ministério da Saúde*-MS), suspected HVL cases should be identified and investigated. Notifications were entered into the SINAN to generate data on epidemiological profiles, mainly with a view to preventive actions, monitoring, and evaluation of risk factors, with the aim of controlling and combating diseases.

Overall, poverty, characterized by inadequate housing, lack of sanitation, and poor solid waste management, is a central factor in facilitating the development and persistence of the visceral leishmaniasis vector. Moreover, unplanned urban expansion, deforestation, and rural-to-urban migration have contributed to the spatial spread of the disease and the formation of new endemic areas. These socio-environmental determinants, which are consistently observed in endemic regions, have enabled the adaptation of the main vector, *Lu. longipalpis* and the presence of domestic dogs as urban reservoirs, particularly in peri-urban settings. Consequently, HVL is not merely a parasitic infection but a complex, environmentally driven disease that is deeply rooted in social inequality and disproportionately affects the most vulnerable populations.

This study had some limitations. First, the predominant use of secondary data from the SINAN entails a potentially high risk of underreporting and inconsistencies in records, which may compromise the accuracy of the epidemiological analysis. Second, the lack of comprehensive studies covering all Brazilian states and absence of municipal-level data, even in the most endemic regions, may introduce bias in representing the true epidemiological panorama of HVL in the country. Finally, the methodological heterogeneity among the reviewed studies, particularly regarding diagnostic criteria and data collection periods, limited the comparability and generalizability of the findings.

Nevertheless, this study provides a comprehensive overview of HVL in Brazil over the past two decades. It highlights the key environmental and sociodemographic factors associated with the persistence and spread of the disease, which may inform future research and public health interventions.

## CONCLUSION

HVL continues to represent a significant public health concern in Brazil. The northeastern region consistently reported the highest incidence rates, whereas the observed expansion of urban transmission across all regions underscores the complexity and dynamism of the epidemiological profile. Although the number of nationally reported cases has recently declined, endemic foci persist and are closely linked to socioenvironmental determinants, such as poverty, unplanned urban growth, deforestation, and inadequate sanitation infrastructure. Accordingly, HVL should be conceptualized not merely as a parasitic disease, but as a reflection of the deep-rooted structural inequalities prevailing within Brazilian society. 

## Data Availability

Data-available-upon-request.
