# Supplementary material for: Human Visceral Leishmaniasis in Brazil in the Past 20 Years: An Epidemiologic Update
Source: Rev Soc Bras Med Trop. 2025 Oct 17;58:e0019-2025. doi: 10.1590/0037-8682-0019-2025 (PMC12534024; doi:10.1590/0037-8682-0019-2025)
Supplement: Supplementary file 1 [file 1678-9849-rsbmt-58-e0019-2025-supp1.pdf]

Supplementary box 1. Search strategy.

Database	Index (English)
Pubmed *	((human visceral leishmaniasis) OR (kala-azar leishmaniasis)) AND (epidemiology) AND (Brazil) NOT (Review)<br>Filter: Language – English; Year: 2002-2022
BVS (Lilacs and Medline)*	((human visceral leishmaniasis) OR (kala-azar leishmaniasis)) AND (epidemiology) AND (brazil) AND NOT (Review)<br>Filter: Language – English; Year: 2002-2022; Database – Medline and Lilacs
Scielo*	((human visceral leishmaniasis) OR (kala-azar leishmaniasis)) AND (epidemiology) AND (brazil) AND NOT (Review)<br>Filter: Language – English; Year: 2002-2022
**Database**	**Index (Portuguese)**
Pubmed	((leishmaniose visceral humana) OR (calazar)) AND (epidemiologia)) AND (Brasil)<br>Filter: Language – Portuguese; Year: 2002-2022
BVS (Lilacs and Medline)	(leishmaniose visceral humana) OR (calazar) AND (epidemiologia) AND (brasil)<br>Filter: Language – Portuguese; Year: 2002-2022; Database – Medline and Lilacs
Scielo	((leishmaniose visceral humana) OR (calazar)) AND (epidemiologia) AND (brasil)<br>Filter: Language – Portuguese; Year: 2002-2022

*The Boolean operators 'NOT' and 'AND NOT' were only used in English searches, as they had little or no effect on Portuguese search results.
